# Fe-CGS Effectively Inhibits the Dynamic Migration and Transformation of Cadmium and Arsenic in Soil

**DOI:** 10.3390/toxics12040273

**Published:** 2024-04-06

**Authors:** Hongliang Yin, Changzhi Zhou, Junhuan Wang, Mengxue Yin, Zhihao Wu, Ningning Song, Xin Song, Yuxian Shangguan, Zaijin Sun, Quanli Zong, Hong Hou

**Affiliations:** 1 School of Resources and Environment, Qingdao Agricultural University, Qingdao 266109, China; yinhongliang97@163.com (H.Y.); snn05@163.com (N.S.); sxin13@163.com (X.S.); 2 State Key Laboratory of Environmental Criteria and Risk Assessment, Chinese Research Academy of Environmental Sciences, Beijing 100012, China; zhouchangzhi1018@163.com (C.Z.); wangjunhuan_1993@163.com (J.W.); yinmengxuer@foxmail.com (M.Y.); wuzh7714@163.com (Z.W.); 3 Institute of Agricultural Resources and Environment, Sichuan Academy of Agricultural Sciences, Chengdu 610066, China; 1987329002@sohu.com; 4 Technical Centre for Soil, Agricultural and Rural Ecology and Environment, Ministry of Ecology and Environment, Beijing 100012, China; szj0007@163.com

**Keywords:** iron modification, coal gasification slag, cadmium, arsenic, leaching, stabilization

## Abstract

The iron-modified coal gasification slag (Fe-CGS) material has excellent performance in purifying heavy-metal-contaminated water due to its good surface properties and adsorption capacities. However, it is unclear whether it can provide long-term simultaneous stabilization of Cd and As in composite-contaminated soils in extreme environments. This study investigated the long-term stabilization of Cd and As in acidic (JLG) and alkaline (QD) soils by simulating prolonged heavy rainfall with the addition of Fe-CGS. Multiple extraction methods were used to analyze the immobilization mechanisms of Cd and As in soil and their effects on bioavailability. The results indicate that the stabilization efficiency was related to the dosage of Fe-CGS. The concentrations of Cd and As in the JLG soil leachate were reduced by 77.6% (2.0 wt%) and 87.8% (1.0 wt%), respectively. Additionally, the availability of Cd and As decreased by 46.7% (2.0 wt%) and 53.0% (1.0 wt%), respectively. In the QD soil leachate, the concentration of Cd did not significantly change, while the concentration of As decreased by 92.3% (2.0 wt%). Furthermore, the availability of Cd and As decreased by 22.1% (2.0 wt%) and 40.2% (1.0 wt%), respectively. Continuous extraction revealed that Fe-CGS facilitated the conversion of unstable, acid-soluble Cd into oxidizable Cd and acid-soluble Cd. Additionally, it promoted the transformation of both non-specifically and specifically adsorbed As into amorphous iron oxide-bound and residual As. Fe-CGS effectively improved the soil pH, reduced the bioavailability of Cd and As, and blocked the migration of Cd and As under extreme rainfall leaching conditions. It also promoted the transformation of Cd and As into more stable forms, exhibiting satisfactory long-term stabilization performance for Cd and As.

## 1. Introduction

The contamination of agricultural soils by heavy metals and metalloids is a significant ecological and environmental issue. Among them, Cd and As contamination poses one of the primary challenges faced by many countries in the environmental sector [1,2]. These contaminants can enter the human body through the food chain and other pathways. Cd and As are toxic to humans and other organisms. Cd can cause serious damage to human kidneys and bones, while As exhibits strong toxicity to most multicellular organisms [3,4]. These elements can be leached out of the soil by rainfall, leading to concentrations in the leachate that exceed regulatory limits. Surface runoff and infiltration can cause the spread of pollution to groundwater and other uncontaminated soil bodies [5,6,7]. To reduce the spread of surface soil Cd and As to groundwater, it is effective to reduce the soluble and exchangeable forms of Cd and As in the soil. Arsenic (As) exists in the form of oxygen-containing anions such as AsO_4_^3−^, and its migration activity increases with increasing pH or decreasing Eh values [8]. Meanwhile, Cd typically exists in soil in cationic form. Its activity is higher in acidic pH ranges. As the soil pH increases, Cd may be adsorbed onto carbonate minerals through diffusion or converted to non-bioavailable Cd(OH)_2_ [9]. The different geochemical behaviors of Cd and As pose a significant challenge to the simultaneous stabilization of both elements in the soil [10,11].

In situ stabilization remediation techniques have become popular methods for remediating soil heavy metal pollution due to their low cost, widespread applicability, and high efficiency [1]. Carbon-based materials are commonly used to stabilize Cd and As in agricultural soil due to their abundant porous structure and functional groups. However, few materials are capable of providing long-term stabilization while efficiently stabilizing both Cd and As. For example, biochar surfaces can carry both positive and negative charges, providing high cation exchange and adsorption capacity. Carbon-based materials have a large specific surface area and abundant active functional groups, allowing for the physical and chemical adsorption of heavy metals in the soil. This alters the specific chemical forms of the heavy metals and inhibits their reactivity and bioavailability. As a result, they are considered high-quality materials for stabilizing soil Cd [12,13,14,15]. Iron-based remediation materials, as environmentally friendly materials, are widely used for the remediation of As-contaminated soil. For instance, nano-zero-valent iron is an ideal material for stabilizing As, possessing high reactivity and abundant surface active sites. It can form stable complexes with As through chelation and other interactions [16,17].

Coal gasification slag (CGS) is a solid residue produced after coal is burned and gasified under high temperatures and high pressure, and it is an industrial by-product emitted during production in the coal chemical industry. Its main components are SiO_2_, Al_2_O_3_, CaO, Fe_2_O_3_, and residual carbon, and its production reaches 30 million tons/year in China [18]. The primary disposal method for CGS currently involves landfilling in mining areas or landfills, which can have negative impacts on the environment and land resources. Research has shown that CGS-based materials exhibit excellent adsorption properties for crystal violet, methylene blue, and some volatile organic pollutants [19,20]. Furthermore, CGS-based materials can purify water bodies contaminated with heavy metals. CGS-based zeolite achieves a removal rate of over 90% for Ni^2+^ in water. CGS-based porous carbon materials are also effective in adsorbing Pb^2+^ from water [18,21,22]. Additionally, the high carbon content, high pH, and abundance of functional groups with negative charges make CGS an ideal material for Cd adsorption, capable of effectively reducing the activity of Cd [23]. Therefore, the preparation of iron-modified CGS (Fe-CGS) provides a new approach to simultaneously stabilize Cd and As in agricultural soil. This method also allows for the utilization of industrial by-products, which is beneficial to reducing environmental pollution and alleviating ecological pressure. The use of Fe-CGS can efficiently immobilize Cd and As in soil, showing tremendous potential for reducing their bioavailability [11].

After stabilization treatments, the release mechanisms of Cd and As in polluted soil mainly involve surface leaching or a combination of surface leaching and migration [24,25]. The primary factor that accelerates the release of Cd and As from stabilized soil matrices is prolonged, high-intensity leaching or an excessively high soil moisture content. Studies have shown that heavy metal leaching concentrations gradually increase with an extended erosion time, leading to a continuous decline in stabilization efficiency and an increased risk of heavy metal migration [26]. Currently, the research on Fe-CGS has primarily focused on the static adsorption of Cd and As. However, there is a lack of relevant studies on whether Fe-CGS exhibits significant dynamic inhibition of the migration and transformation of Cd and As in soil under extreme conditions such as high-intensity leaching and erosion. Therefore, further research is needed.

In this study, soil-column-simulated experiments were conducted to investigate the long-term effects of Fe-CGS on inhibiting the migration of Cd and As in soil through simulated heavy rainfall leaching. This study also investigated the changes in the forms of Cd and As to understand how Fe-CGS inhibits their migration and transformation. This research aimed to develop strategies to mitigate the leaching of Cd and As from soil, which can lead to groundwater pollution and diffusion, particularly during heavy rainfall. Moreover, this study introduces a new method of using industrial by-products on agricultural land, which can stabilize the balance between commercial benefits and environmental protection and, thus, explore the prerequisites for achieving green and circular development.

## 2. Materials and Methods

### 2.1. Experimental Materials

Fe-CGS was synthesized in our laboratory, composed of C (68.6%, proportion by weight), O (21.34%), K (2.17%), and Fe (5.79%). Fe-CGS can simultaneously adsorb Cd and As from a solution, with adsorption capacities of up to 42.72 mg/g and 35.29 mg/g, respectively [11].

Experimental soil samples were collected from Qingduo (QD) (112°29′25″ E, 35°10′0.4″ N) and Jiuligou (JLG) (112°25′54″ E, 35°10′46″ N) in Henan Province, China. Both are surface soils (0–20 cm) from farmland, with soil pH values of 7.77 and 5.27, respectively. The soils were highly contaminated with Cd. To simulate the combined pollution with Cd and As, exogenous As was added to the soil. An appropriate amount of Na_2_HAsO_4_·7H_2_O solution was sprayed onto the soil and thoroughly mixed. Distilled water was added to adjust the soil moisture to 40% of the field capacity. The mixed soil was placed in open plastic bags and aged at a constant temperature of 25 °C for 30 days. During aging, the soil was re-watered every 5 days to maintain the soil moisture at 40% of the field capacity. The Cd content in the QD soil was 7.79 mg/kg, and the As content was 87.05 mg/kg. The Cd content in the JLG soil was 2.82 mg/kg, and the As content was 72.19 mg/kg.

### 2.2. Experimental Design

In this experiment, soil column leaching was used to simulate the effect of Fe-CGS on the release characteristics and migration behaviors of Cd and As in agricultural soil under conditions of excessive soil moisture during extreme rainfall.

#### 2.2.1. Soil Column Packing

Soil samples of 100.000 g were accurately weighed in a beaker, and Fe-CGS was added in mass percentages. The soil samples were divided into three treatments according to the percentage of Fe-CGS added, and three replicates were set up for each treatment: G0 (0 wt%), G1 (1.0 wt%), and G2 (2.0 wt%). The Fe-CGS and soil samples were thoroughly mixed well with a glass rod.

The mixtures of experimental soil and Fe-CGS were packed into Plexiglas tubes of a 15 cm height and 3 cm inner diameter. The soil columns were filled from bottom to top in the following order: a 0.1 mm thick layer of nylon mesh, a 1 cm thick layer of glass beads (3 mm in diameter), a 1 cm thick layer of quartz sand, the mixture of a soil sample and Fe-CGS, and another 1 cm thick layer of quartz sand.

#### 2.2.2. Leaching

**Figure 1:**
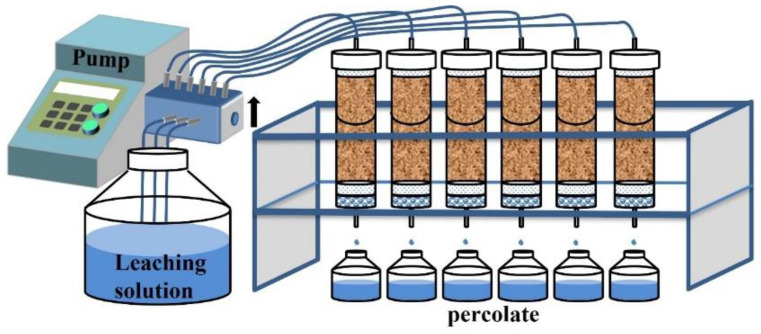
Schematic diagram of leaching experimental apparatus.

Deionized water was injected from the bottom outlet using a peristaltic pump (Rongbai, Baoding, China) to saturate the soil column, and then it was allowed to equilibrate for 7 days (Figure 1). After equilibration, continuous leaching was conducted by adding a leachate solution from the top to the bottom of the soil column at a constant rate (0.38 mL/min) using a peristaltic pump. The leaching process continued for 144 h, using a total of 3283.2 mL of the leachate solution, simulating a rainfall of approximately 4647 mm. The leachate solution was prepared using deionized water obtained from a water purification system (HHitech, Shanghai, China). A collection bottle for the leachate was placed at the bottom of the soil column, with collection intervals set at 24 h. The collected leachate samples were filtered through a 0.45 μm hydrophilic filter membrane and stored in centrifuge tubes. The concentrations of Cd and As in the leachate were determined using ICP-MS (Agilent, Santa Clara, CA, USA).

### 2.3. Determination of Effective Forms of Cd and As

After the leaching experiment, the mixtures of soil and Fe-CGS in the soil columns were air-dried for the determination of the effective forms of Cd and As.

The effective form of Cd in the soil was extracted using 0.10 M CaCl_2_. An amount of 1.000 g of the mixed sample was accurately weighed into a 50 mL hard glass triangular flask (centrifuge tube), and 25 mL of the 0.1 M CaCl_2_ solution was added. The ratio of soil to solution was 1:25. The flask was placed on a horizontal reciprocating shaker at room temperature (25 ± 2 °C) and oscillated at a speed of 180 r/min for 2. It was centrifuged at 3000 r/min for 10 min, and then the supernatant was taken and filtered through a 0.45 μm filter membrane. The concentration of effective Cd in the filtrate was determined by ICP-MS (Agilent, Santa Clara, CA, USA).

The effective form of As in the soil was extracted using 0.5 M NaHCO_3_. An amount of 1.000 g of the air-dried mixed sample was accurately weighed into a 50 mL hard glass triangular flask (centrifuge tube), and 25 mL of the 0.5 M NaHCO_3_ solution was added. The flask was placed on a horizontal reciprocating shaker at room temperature (25 ± 2 °C) and oscillated at a speed of 180 r/min for 2 h. It was centrifuged at 3000 r/min for 10 min, and then the supernatant was taken and filtered through a 0.45 μm filter membrane. The concentration of effective As in the filtrate was determined by ICP-MS (Agilent, Santa Clara, CA, USA).

### 2.4. Analysis of Cd and As Fractions

The BCR method was applied to analyze the chemical forms of Cd in the mixed samples, which were divided into weak-acid-extractable Cd (F1), reducible Cd (F2), oxidizable Cd (F3), and residual Cd (F4). The required amount of a mixed soil sample for this method was 0.500 g. The extraction agents and methods for each form were as follows: 20 mL of a 0.11 mol/L acetic acid solution, shaken for 16 h (180 rpm, 25 °C); 20 mL of a 0.5 mol/L NH_2_OH·HCl solution, shaken for 16 h (180 rpm, 25 °C); 10 mL of 30% H_2_O_2_ (pH = 2~3) and 25 mL of 1 mol/L NH_4_OAc (pH = 2), with 30% H_2_O_2_ added twice, 5 mL each time, and digestion in a water bath for 2 h (85 ± 2 °C); and 2 mL of HNO_3_/6 mL of a HCl mixed solution, digested until the solution turned transparent (heated in three stages: 100 °C for 2 min, 150 °C for 3 min, and 180 °C for 25 min). After each extraction step, the supernatant was collected by centrifugation (4000 rpm) for 10 min and filtered.

We employed the Wenzel method outlined in the literature [27] to analyze the forms of As in the mixed samples, including the non-specifically adsorbed As fraction, specifically adsorbed fraction, amorphous iron oxide-bound As fraction, crystalline iron oxide-bound As fraction, and residual As fraction. The required amount of a mixed soil sample for this method was 1.000 g. The extraction agents and methods for each form were as follows: 25 mL of 0.05 M (NH_4_)_2_SO_4_, shaken for 4 h (180 rpm, 25 °C, in darkness); 25 mL of 0.05 M NH_4_H_2_PO_4_, shaken for 16 h (180 rpm, 25 °C, in darkness); 25 mL of 0.2 M NH_4_^+^-oxalate (pH = 3.25), shaken for 4 h (180 rpm, 25 °C, in darkness); and 25 mL of 0.2 M NH_4_^+^-oxalate + 0.1 M ascorbic acid (pH = 3.25, 96 °C, 0.5 h), with the residual residue dissolved in HNO_3_ + H_2_O_2_ until transparent. After each extraction step, the supernatant was collected by centrifugation (4000 rpm) for 10 min and filtered.

The reagents used in each step of the BCR and Wenzel methods were of analytical grade, and the extracted solution was filtered through a 0.45 μm filter membrane (JIN TENG, Tianjin, China), and then the concentrations of Cd and As in the filtrate were determined by ICP-MS (Agilent, Santa Clara, CA, USA).

## 3. Results

### 3.1. Changes in Soil Leachate pH

**Figure 2:**
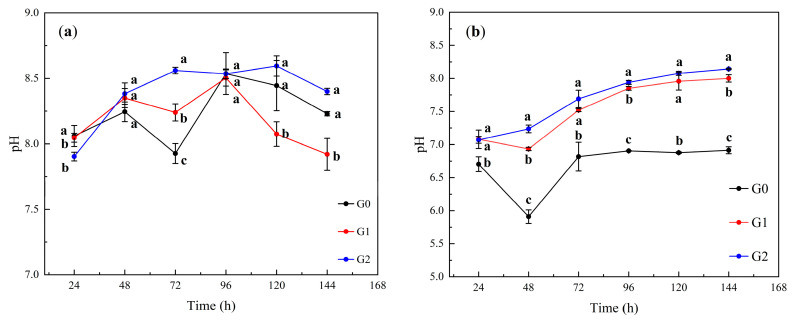
Soil leachate pH. (**a**): Soil leachate pH of QD (alkaline) soil. (**b**): Soil leachate pH of JLG (acidic) soil. G0, G1, and G2 represent the addition of Fe-CGS at concentrations of 0.0 wt%, 1.0 wt%, and 2.0 wt%, respectively. Error bars represent the standard deviation of the mean (*n* = 3). Different letters indicate significant differences (*p* < 0.05).

The soil pH is one of the primary factors affecting the activities of Cd and As. The variation in the soil leachate pH with the leaching duration for QD is depicted in Figure 2a. At 24 h, the leachate pH of the G2 (7.90) treatment group was lower than that of G0 (8.06) and G1 (8.05), but the leachate pH of the G2 treatment group was higher than that of G0 and G1 when the leaching time was greater than 48 h (*p* < 0.05). The pH of the soil leachate in the G0, G1, and G2 treatment groups was 8.23, 7.92, and 8.4, respectively, for the duration of the experiment up to 144 h. Therefore, in alkaline soils, the greatest effect on the soil leachate pH was observed when Fe-CGS was added at a rate of 2.0 wt%.

The variation in the soil leachate pH with the leaching duration for JLG is depicted in Figure 2b. At 24 h, the soil leachate pH of the G0 treatment group (6.70) was significantly lower than that of G1 (7.08) and G2 (7.08). With the increasing leaching time, the soil leachate pH of the G1 and G2 treatment groups continued to rise and was significantly higher than that of the G0 treatment group (*p* < 0.05). By the end of the experiment at 144 h, the soil leachate pH values for the G0, G1, and G2 treatment groups were 6.91, 8.00, and 8.14, respectively. Thus, the addition of Fe-CGS at a concentration of 2.0 wt% significantly increased the pH of the acidic soil (*p* < 0.05). However, the maximum enhancement was observed at an addition level of 2.0 wt%.

### 3.2. The Stabilizing Effect of Fe-CGS on Cd

**Figure 3:**
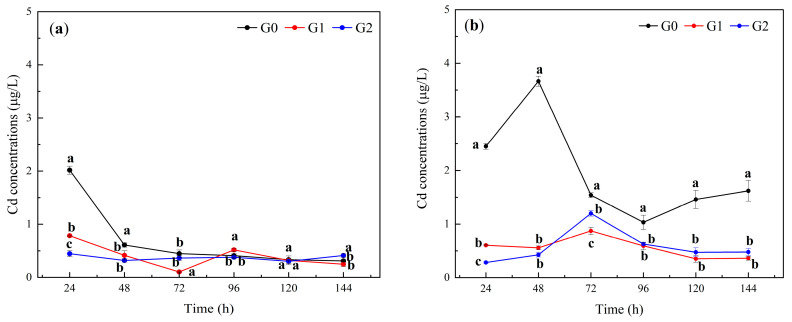
Soil leachate Cd concentrations. (**a**): Cd concentration in QD (alkaline) soil leachate. (**b**): Cd concentration in JLG (acidic) soil leachate. G0, G1, and G2 represent the addition of Fe-CGS at concentrations of 0.0 wt%, 1.0 wt%, and 2.0 wt%, respectively. Error bars represent the standard deviation of the mean (*n* = 3). Different letters indicate significant differences (*p* < 0.05).

The variation in the Cd concentration in the soil leachate of QD is depicted in Figure 3a. During the initial leaching period, the concentration of Cd in the soil leachate treated with Fe-CGS was significantly lower than that in the control group (G0) (*p* < 0.05). After 24 h of leaching, the Cd concentrations in the leachates of the G0, G1, and G2 treatment groups were 2.02 μg/L, 0.78 μg/L, and 0.45 μg/L, respectively, with significant differences observed between each treatment group (*p* < 0.05). Subsequently, the Cd concentrations in the leachates of the G0, G1, and G2 treatment groups continued to decrease and tended to stabilize after 48 h. By the end of the 144 h leaching period, the Cd concentrations in the leachates of the G0, G1, and G2 treatment groups were 0.31 μg/L, 0.25 μg/L, and 0.41 μg/L, respectively. Therefore, Fe-CGS had a stabilizing effect on Cd in alkaline soil at the early stage of leaching, but the stabilizing effect was not significant at the later stage.

The variation in the Cd concentration in the soil leachate of JLG is depicted in Figure 3b. The concentration of Cd in the soil leachate treated with Fe-CGS was significantly lower than that in the control group (G0) (*p* < 0.05). Additionally, the Cd concentration in the leachate of the G0 treatment group remained consistently high. After 24 h of leaching, the Cd concentrations in the leachates of the G0, G1, and G2 treatment groups were 2.45 μg/L, 0.60 μg/L, and 0.28 μg/L, respectively, with significant differences observed between each treatment group (*p* < 0.05). Subsequently, the Cd concentrations in the leachates of the G1 and G2 treatment groups remained relatively stable. By the end of the 144 h leaching period, the Cd concentrations in the leachates of the G0, G1, and G2 treatment groups were 1.62 μg/L, 0.36 μg/L, and 0.48 μg/L, respectively. Fe-CGS exhibited a significant stabilizing effect on Cd in acidic soil, with the stabilization lasting for more than 144 h under intensive leaching conditions.

### 3.3. The Stabilizing Effect of Fe-CGS on As

**Figure 4:**
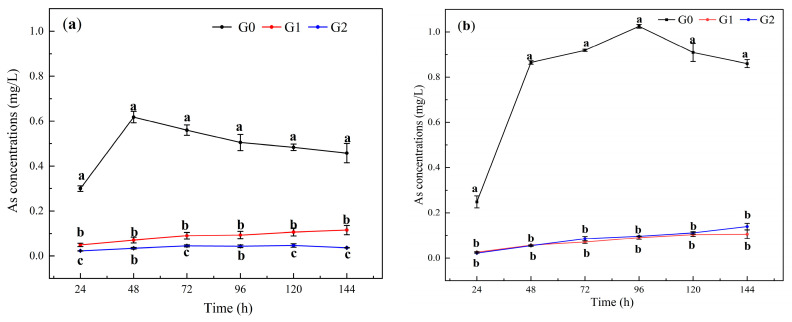
Soil leachate As concentrations. (**a**): As concentration in QD (alkaline) soil leachate. (**b**): As concentration in JLG (acidic) soil leachate. G0, G1, and G2 represent the addition of Fe-CGS at concentrations of 0.0 wt%, 1.0 wt%, and 2.0 wt%, respectively. Error bars represent the standard deviation of the mean (*n* = 3). Different letters indicate significant differences (*p* < 0.05).

The variation in the As concentration in the QD soil leachate with the drenching time is shown in Figure 4a. The As concentration in the Fe-CGS-treated soil leachate was significantly lower than that in the control group (0.0 wt%) (*p* < 0.05). The leachate As concentrations in the G0, G1, and G2 treatment groups were 0.30 mg/L, 0.05 mg/L, and 0.02 mg/L at 24 h of leaching, respectively, and then the leachate As concentrations in the G1 and G2 treatment groups remained relatively stable with the extension of the leaching time. However, the leachate As concentration in the G0 treatment group continuously decreased after 48 h. At the end of drenching, the leachate As concentrations in the G0, G1, and G2 treatment groups were 0.46 mg/L, 0.12 mg/L, and 0.04 mg/L, respectively, and the leachate As concentrations in G1 and G2 were significantly lower than that in G0 (*p* < 0.05). Therefore, the addition of Fe-CGS had a positive effect on the stabilization of As in alkaline soil.

The variation in the As concentration in the JLG soil leachate with the drenching time is shown in Figure 4b. The concentration of As in the Fe-CGS-treated soil leachate was significantly lower than that in the control group (G0) (*p* < 0.05). The As concentrations in the leachates of the G0, G1, and G2 treatment groups were 0.25 mg/L, 0.03 mg/L, and 0.02 mg/L at 24 h, respectively. Then, the As concentrations in the leachates of the G1 and G2 treatment groups continuously increased with the extension of the leaching time, and the maximum values were 0.10 and 0.14 mg/L, respectively. The concentration of the leachate in the G0 treatment group continued to increase to 1.02 mg/L, then decreased slightly, and finally remained at 0.86 mg/L. Therefore, the addition of Fe-CGS to acidic soil could enhance the stabilization of As and reduce the risk of diffusion.

### 3.4. Changes in the Available of Cd and As

**Figure 5:**
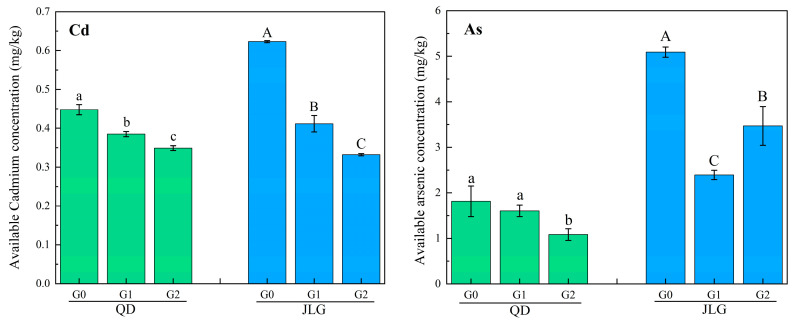
Contents of effective Cd and As states in co-polluted soils after leaching experiments. QD and JLG denote alkaline and acidic soils, respectively. G0, G1, and G2 denote the additions of 0.0 wt%, 1.0 wt%, and 2.0 wt% of Fe-CGS, respectively. Error bars indicate the standard deviation of means (*n* = 3), and different letters indicate significant differences (*p* < 0.05).

The available Cd and As in the QD and JLG soils after continuous leaching under simulated extreme rainfall conditions are shown in Figure 5. The available Cd in the Fe-CGS-treated group of QD soils was significantly lower than that in the control group (G0) (*p* < 0.05) and showed a tendency to decrease with the increase in the amount of Fe-CGS added. The available Cd in the soil was 0.45 mg/kg, 0.39 mg/kg, and 0.35 mg/kg with the addition of 0.0 wt%, 1.0 wt%, and 2.0 wt% of Fe-CGS, respectively, and the available Cd in the soil was reduced by 14.0% and 22.1% with the addition of 1.0 wt% and 2.0 wt% of Fe-CGS. The available state contents of As were 1.81 mg/kg, 1.60 mg/kg, and 1.08 mg/kg when Fe-CGS was added at the levels of G0, G1, and G2, respectively. The available state contents of As decreased by 11.6% and 40.2% with the increase in the addition of Fe-CGS compared with the control group, respectively. When the addition of Fe-CGS was increased to 2.0 wt%, the available state content of As was significantly lower than that in the G0 and G1 groups (*p* < 0.05). Therefore, Fe-CGS still maintained a strong ability to immobilize the available Cd and As in alkaline soil after high-intensity leaching. Higher Fe-CGS addition was beneficial for improving the stabilization effect, so it is recommended to increase the addition amount of Fe-CGS without exceeding the limit value.

The available Cd in the JLG soil treated with Fe-CGS (G1 and G2) was significantly lower (*p* < 0.05) than that in the control (G0), and the available Cd was strongly influenced by Fe-CGS. The available Cd was 0.62 mg/kg, 0.41 mg/kg, and 0.33 mg/kg in G0, G1, and G2, respectively. The available Cd decreased by 33.9% and 46.7% with 1.0 wt% and 2.0 wt% addition of Fe-CGS. The available As was significantly affected by the Fe-CGS treatment of contaminated soil. The available As levels were 5.09 mg/kg, 2.39 mg/kg, and 3.47 mg/kg in G0, G1, and G2, respectively. The available As was reduced by 53.0% with 1.0 wt% Fe-CGS, while it was reduced by only 31.8% when the dosage of Fe-CGS increased to 2.0 wt%. Therefore, increasing the dosage of Fe-CGS was beneficial for reducing the availability of Cd in acidic soil, but the continuous increase in the dosage was unfavorable for reducing the availability of As.

### 3.5. Changes in Cd and As Fractions

**Figure 6:**
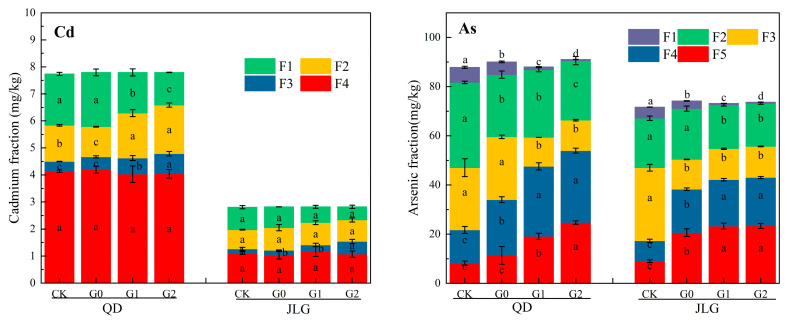
Fraction analysis of Cd and As in co-polluted soil. QD and JLG denote alkaline and acidic soil, respectively. G0, G1, and G2 denote the addition of Fe-CGS at 0.0 wt%, 1.0 wt%, and 2.0 wt%, respectively. CK denotes the content of different forms of Cd and As in the unleached soil. Error bars indicate standard deviation of means (*n* = 3), and different letters indicate significant differences (*p* < 0.05).

The effects of Fe-CGS on the morphologies of Cd and As in acidic and alkaline co-polluted soils before and after leaching were investigated using the BCR continuous extraction method and the Wenzel five-step extraction method, respectively (Figure 6).

Cd in the control (CK) QD soil was dominated by the weak-acid-extractable (F1) and residual (F4) states, with contents of 1.90 mg/kg and 4.13 mg/kg, respectively. The reducible (F2) and oxidizable (F3) states had lower contents of 1.34 mg/kg and 0.36 mg/kg, respectively. The content of Cd (F1) in the weak-acid-extractable state significantly decreased (*p* < 0.05) with the increase in Fe-CGS addition. When Fe-CGS was added at 1.0 wt% and 2.0 wt%, its content decreased to 1.51 mg/kg and 1.21 mg/kg, respectively. The content of the residual state Cd (F4) in the soil after Fe-CGS treatment did not show any significant difference compared with CK. When the Fe-CGS addition was increased to 2.0 wt%, the contents of the reducible state Cd (F2) and oxidizable state Cd (F3) were significantly increased compared with CK. Non-specialized adsorbed As (F1), specialized adsorbed As (F2), and the amorphous Fe oxide-bound state As (F3) in the soil significantly decreased (*p* < 0.05) with the increase in Fe-CGS addition and were 0.49 mg/kg, 24.2 mg/kg, and 12.41 mg/kg when Fe-CGS was added at 2.0 wt%, respectively. The content of the crystalline Fe oxide-bound state As (F4) and residue state As (F5) significantly increased (*p* < 0.05) with the increase in Fe-CGS addition, and their contents were 29.24 mg/kg and 24.71 mg/kg when the Fe-CGS addition was increased to 2.0 wt%, respectively. The alkaline QD soils treated with Fe-CGS were still effective in reducing the contents of the weak-acid-extractable state Cd and non-specialized adsorbed As, which are the most active fractions of Cd and As.

After Fe-CGS treatment, the content of weak-acid-extractable Cd (F1) in the JLG soil gradually decreased with the increase in Fe-CGS addition, and the content was 0.48 mg/kg at the maximum addition of Fe-CGS (2.0 wt%); however, the content of reducible Cd (F2) and oxidizable Cd (F3) gradually increased, and their contents were 0.80 mg/kg and 0.47 mg/kg at an Fe-CGS addition amount of 2.0 wt%, respectively. There was no significant difference (*p* < 0.05) in the residual state Cd (F4) content in the treatment group compared with CK. In addition, the non-specialized adsorbed As (F1), specialized adsorbed As (F2), and amorphous iron oxide-bound As (F3) significantly decreased (*p* < 0.05) with the increase in Fe-CGS addition. Moreover, their contents were lowest when Fe-CGS was added at 2.0 wt%, and were 0.49 mg/kg, 24.20 mg/kg, and 12.41 mg/kg, respectively. The crystalline iron oxide-bound state As (F4) and residue state As (F5) significantly increased (*p* < 0.05) with the increase in Fe-CGS addition and were 29.34 mg/kg and 24.71 mg/kg when the Fe-CGS addition was increased to 2.0 wt%. Fe-CGS had a positive effect on stabilizing Cd in the JLG soil (acidic) under high-intensity leaching conditions. It effectively reduced the content of its active component. For As, Fe-CGS both effectively reduced the content of the active component and promoted the generation of As in its residual state, which was conducive to the immobilization of As in the soil.

## 4. Discussion

### 4.1. Changes in Soil pH

The pH affects the activity and fugitive forms of Cd and As. With the addition of Fe-CGS, the soil pH was significantly increased, which promotes the fixation of Cd by soil particles. Under a higher pH, most of the soil particles and passivated materials are negatively charged due to the deprotonation of functional groups, such as phenol, hydroxyl, and carboxyl groups, which is beneficial for the adsorption of cations like Cd [28]. However, As is predominantly in the negatively charged free state, and the bioavailability of As increases as the soil pH rises [29]. In this study, the release of alkaline cations such as K^+^ and Ca^2+^ from Fe-CGS resulted in a higher leachate pH in the treatment group than in the control group, and the pH maintained a continuous upward trend in the acidic JLG soil. Meanwhile, the dissolution process of alkaline substances such as calcium hydroxide and hydrated calcium silicate in the soil also contributed to the increase in the soil leachate pH, and a significant increase in the soil pH was also observed in a study using alkaline biocarbon to co-stabilize soil Cd and As [29,30,31]. However, there was no significant difference (*p* < 0.05) in pH change in alkaline QD soils. Therefore, Fe-CGS increased the pH of acidic soils and maintain a stabilizing trend over a long period of time during leaching but had no significant effect on alkaline soils.

### 4.2. Leaching Behavior and Availability of Cd and As

The addition of Fe-CGS could affect the leaching characteristics of Cd and As in soil. Moreover, it can significantly reduce the availability of Cd and As. The Cd content of leachate in the treatment group was consistently maintained in a low range because of the deprotonation of acidic functional groups and the soil, where the soil particles and Fe-CGS were more negatively charged, which resulted in showing a stronger electrostatic affinity for positively charged Cd^2+^ [32]. Meanwhile, a continuous increase in pH induced the gradual conversion of Cd into the more stable Cd(OH)_2_. It has been shown that Cd(OH)_2_ occupies the highest percentage in the system at a pH of > 8 [33]. In addition, −OH in Fe-CGS can form [(MOH)]^n+^ with Cd, which further enhances the surface complexation and reduces the bioavailability of Cd while facilitating the conversion of soluble Cd(II) to Cd(OH)_2_. Cd(OH)_2_ is a much more stable and unavailable form in soils, and with a sustained increase in pH, Cd(OH)_2_ can be hydrolyzed to [Cd(OH)]^+^ and interacts with Fe-OH on the surface of Fe-CGS by co-precipitation to form a stabilized product [16,34]. Cd migration from a soil solution to the Fe-CGS surface is driven by electrostatic attraction [11]. Meanwhile, Fe-CGS can produce hydroxides on the FeOOH surface during the preparation of iron oxides, providing surface sites for Cd adsorption [32]. This adsorption process is similar to the adsorption of metal ions on Fe [35] oxides (e.g., acicular ferrite, hydrated iron, and magnetite) due to the ability of Cd to be partially adsorbed or complexed by Fe [35] minerals [36].

The oxide and hydroxide groups of Fe in Fe-CGS play a decisive role in the stabilization of As. As can form stable Fe-O-As complexes on the surfaces of Fe-containing materials, and Fe complexation with As on the surfaces of materials is the main mechanism for stabilizing As [1,11,37]. Because, as shown by the characterization results, -OH, -CH, C=O, and C=C/C≡C in Fe-CGS are not only retained but also strengthened, and at the same time, due to the modification to generate FeO, these functional groups are used as effective adsorption sites for As [38].

As in soil can be adsorbed exclusively on the surface of Fe_2_O_3_ in Fe-CGS, which is the main reason for reducing the bioavailability of As. It has been shown that Fe_2_O_3_ has a strong affinity for As, which can be easily adsorbed by Fe_2_O_3_ in the form of monodentate-binding compounds [39,40]. In addition, oxygen atoms on -OH and C=O can provide free electron pairs to interact with ions of As [41]. In this process, adsorption, co-precipitation, and complexation are the main mechanisms for immobilizing As and are essential for stabilizing and controlling the diffusion of As in soils [42].

Compared with alkaline soil (QD), Fe-CGS has a strong blocking effect on Cd in acidic soil (JLG), and the electrostatic attraction, surface complexation, and co-precipitation of Cd by Fe-CGS are the main reasons for preventing the diffusion of high concentrations of Cd with the leachate. Fe-CGS has a strong blocking and controlling ability for the diffusion of As in both acidic and alkaline soils, and the blocking time is longer than 144 h in high-intensity leaching. Fe-CGS can reduce the bioavailability of Cd, and -OH is involved in the formation of hydroxylated metal ions, and complexation and electrostatic attraction are also important mechanisms for reducing the bioavailability, while the effect of Fe-CGS on the effective state content of As is mainly dependent on the surface-specific adsorption of As by Fe_2_O_3_.

### 4.3. Cd and As Fractions

Fe-CGS promoted the transformation of the F1 component of Cd to F2 and F3 in alkaline soil (QD) and acidic soil (JLG), so that Cd was transformed from the more active weak-acid-extractable state to the more stable reducible and oxidizable states, and the degree of transformation was enhanced with the addition of Fe-CGS. During the continuous high-intensity leaching process, Cd was desorbed from soil particles and diffused to the surface of Fe-CGS and was adsorbed by the active sites, and the products formed were mainly FeOCd^+^ and FeOCdOH. Fe-CGS releases alkaline cations as well as OH^−^, which increases the soil pH as one of the important factors promoting Cd precipitation, and Fe^2+^ produced during long-duration leaching may co-precipitate with Cd on the surfaces of iron minerals to form stable minerals [43]. CaCO_3_ in Fe-CGS also allows soluble Cd to be deposited as Cd(OH)_2_ on the Fe-CGS surface [11]. The abundance of oxygen-containing functional groups, such as carboxyl and hydroxyl groups, on the surface of Fe-CGS is also a major factor that promotes the conversion of F1 (the weak-acid-extractable state) to F3 (the oxidizable state) [44,45].

Fe in Fe-CGS is the main factor in stabilizing soil As. The F1 and F2 fractions of As in soil are considered to be the most mobile parts of the soil, and the bioavailability of As is significantly correlated with the concentrations of the F1 and F2 fractions. It was found that the As content in brown rice and straw was significantly and positively correlated with the concentrations of the F1 and F2 fractions of As in the soil, so the F1 and F2 fractions of As can be considered to be the plant-available fractions of As in the soil [46,47]. For the present study, the contents of the F1 and F2 fractions significantly decreased with increasing Fe-CGS addition. The F1 and F2 fractions in acidic soil (QD) accounted for 1–7% and 27–39% of the total As, respectively.

F1 and F2 in alkaline soil (JLG) accounted for 1–6% and 24–28% of the total As, respectively. The F3, F4, and F5 fractions are less bioavailable and difficult to be absorbed by plants, e.g., As in brown rice and straw is significantly negatively correlated with the F3 fraction of As in the soil, and there is no significant correlation with the F4 and F5 fractions [46]. Therefore, the effective reduction in the F1 and F2 contents is a prerequisite for reducing As biotoxicity. Increasing the content of amorphous iron oxides (Fe^3+^) can promote the F1 and F2 components of As to the F3 component. The large increase in the F5 (the residual state) content in the soil was mainly related to the adsorption and precipitation of Fe-CGS, and the adsorption of As on the surfaces of stabilized materials was mainly determined by the charge of the adsorbed surface. A large number of iron-containing functional groups, such as FeOH^2+^, FeOH, and FeO^−^, tend to form on the surface of Fe-CGS during the preparation process. And the As in the soil solution is predominantly HAsO_4_^2−^, so the interaction of HAsO_4_^2−^ with the positively charged functional groups of Fe-CGS promotes the adsorption of As. Meanwhile, the release and hydrolysis of Fe^3+^ in the material can form stable Fe-As coprecipitation compounds with As, which is also an important As stabilization mechanism [48,49]. The application of Fe-CGS significantly reduced the content of the acid-soluble form fraction of Cd in the soil, with no significant difference in the content of the residual form. For As, it significantly reduced the content of unstable form fractions and simultaneously converted them into stable residue state fractions and increased with the increase in Fe-CGS addition. Among them, the type and concentration of Fe are important factors affecting the distribution ratio of As components.

## 5. Conclusions

In this study, the ability of Fe-CGS to block the vertical migration of Cd and As during leaching was further explored on the basis of the ability of Fe-CGS to statically stabilize Cd and As, which has been demonstrated in previous work. The results show that Fe-CGS could significantly enhance the pH of the soil leachate and, at the same time, significantly reduce the concentrations of Cd and As in the leachate with the extension of the simulation time. The available Cd and As in the soil significantly decreased with the increase in Fe-CGS. Fe-CGS had a strong stabilizing ability for As in acidic and alkaline soils under high-intensity leaching, but it was difficult to achieve the long-term blocking and controlling of the release of Cd in alkaline soils. Cd was mainly stabilized by Fe-CGS via complexation, while As was immobilized via adsorption, co-precipitation, and complexation. Cd in the soil was converted from unstable components to reducible and oxidizable states, and As was converted to the Fe-bound form and residue state. Fe-CGS has the potential to continuously block and stabilize Cd and As in soils, even under long-term extreme rainfall or high levels of Cd and As pollution. This indicates that CGS, an industrial by-product, could be used as a restorative material in agriculture.

## Data Availability

The data presented in this study are available upon request from the corresponding author.
